# Anti-proteinase 3 Antibody (PR3)-Anti-neutrophil Cytoplasmic Antibody (ANCA)-Positive Vasculitis: A Rare Presentation in a 90-Year-Old Female

**DOI:** 10.7759/cureus.97341

**Published:** 2025-11-20

**Authors:** Sara Shehab, Hesham K Badr, Hammad Khan, Sohaib Eladl, Mohamed Taha

**Affiliations:** 1 Internal Medicine, Blackpool Teaching Hospitals NHS Foundation Trust, Blackpool, GBR; 2 Geriatrics, Internal Medicine, Blackpool Teaching Hospitals NHS Foundation Trust, Blackpool, GBR; 3 Geriatrics, Blackpool Teaching Hospitals NHS Foundation Trust, Blackpool, GBR; 4 Acute Medicine, Blackpool Teaching Hospitals NHS Foundation Trust, Blackpool, GBR

**Keywords:** acute kidney injury, autoimmune, c-anca, c-anca/proteinase 3-positive granulomatosis with polyangiitis, canca/ proteinase 3 (pr3)-positive granulomatosis with polyangiitis (gpa) (formerly known as wegener's granulomatosis, geriatric medicine, granulomatosis with polyangiitis (gpa), haemoptysis, microscopic polyangiitis, vasculitis

## Abstract

Granulomatosis with polyangiitis (GPA), a rare systemic vasculitis, typically affects middle-aged adults, with incidence declining significantly in the elderly. Here, we present a rare and exceptional case of new-onset GPA in a 90-year-old female presenting with hemoptysis and characteristic systemic manifestations. Initially investigated for suspected lung malignancy due to her advancing age and history of non-Hodgkin’s lymphoma (in remission since 2017), the patient was diagnosed with proteinase 3 (PR3) anti-neutrophil cytoplasmic antibody (ANCA)-positive vasculitis and characteristic imaging findings.

Treatment with corticosteroids, immunosuppressants, and avacopan resulted in improved renal function and overall resolution of symptoms, highlighting the importance of considering vasculitis as a differential diagnosis in elderly patients with multisystem involvement. This case report features the diagnostic challenges and therapeutic considerations of late-onset GPA in the geriatric population.

## Introduction

Formerly known as Wegener’s granulomatosis, granulomatosis with polyangiitis (GPA) is a rare anti-neutrophil cytoplasmic antibody (ANCA)-associated vasculitis characterized by necrotizing granulomatous inflammation of small- and medium-sized vessels. The classic clinical triad for GPA involves the upper respiratory tract (sinusitis, nasal crusting, saddle-nose deformity), the lower respiratory tract (nodules, cavities, alveolar hemorrhage), and the kidneys (rapidly progressive glomerulonephritis); however, constitutional symptoms and multi-organ involvement are also common [1]. In the United Kingdom, the incidence of GPA is 11.8 cases per million person-years, with a clear peak between 55 and 69 years [2]. In older patients, particularly those over 80 years of age, the diagnosis of GPA is uncommon, and microscopic polyangiitis (MPA) is more frequently observed. In fact, in the geriatric population, late-onset GPA presents distinctive difficulties - its clinical presentation often diverges from the classic profile, and the presence of multiple comorbidities further complicates both recognition and treatment [3,4].

Proteinase 3 (PR3)-ANCA is detected in approximately 90% of cases of GPA, helping to distinguish it from other ANCA-associated vasculitides. For example, MPA is typically myeloperoxidase (MPO)-ANCA positive and lacks granulomatous inflammation, while eosinophilic GPA (EGPA) shows ANCA positivity in only around 30%-40% of cases (when positive, mostly MPO) and is associated with asthma and eosinophilia [5,6].

We present a case of new-onset PR3-ANCA-positive vasculitis with rapidly progressive multisystem involvement in a 90-year-old woman, emphasizing the need for heightened suspicion of autoimmune vasculitis even in elderly patients.

## Case presentation

Clinical history

**Figure 1:**
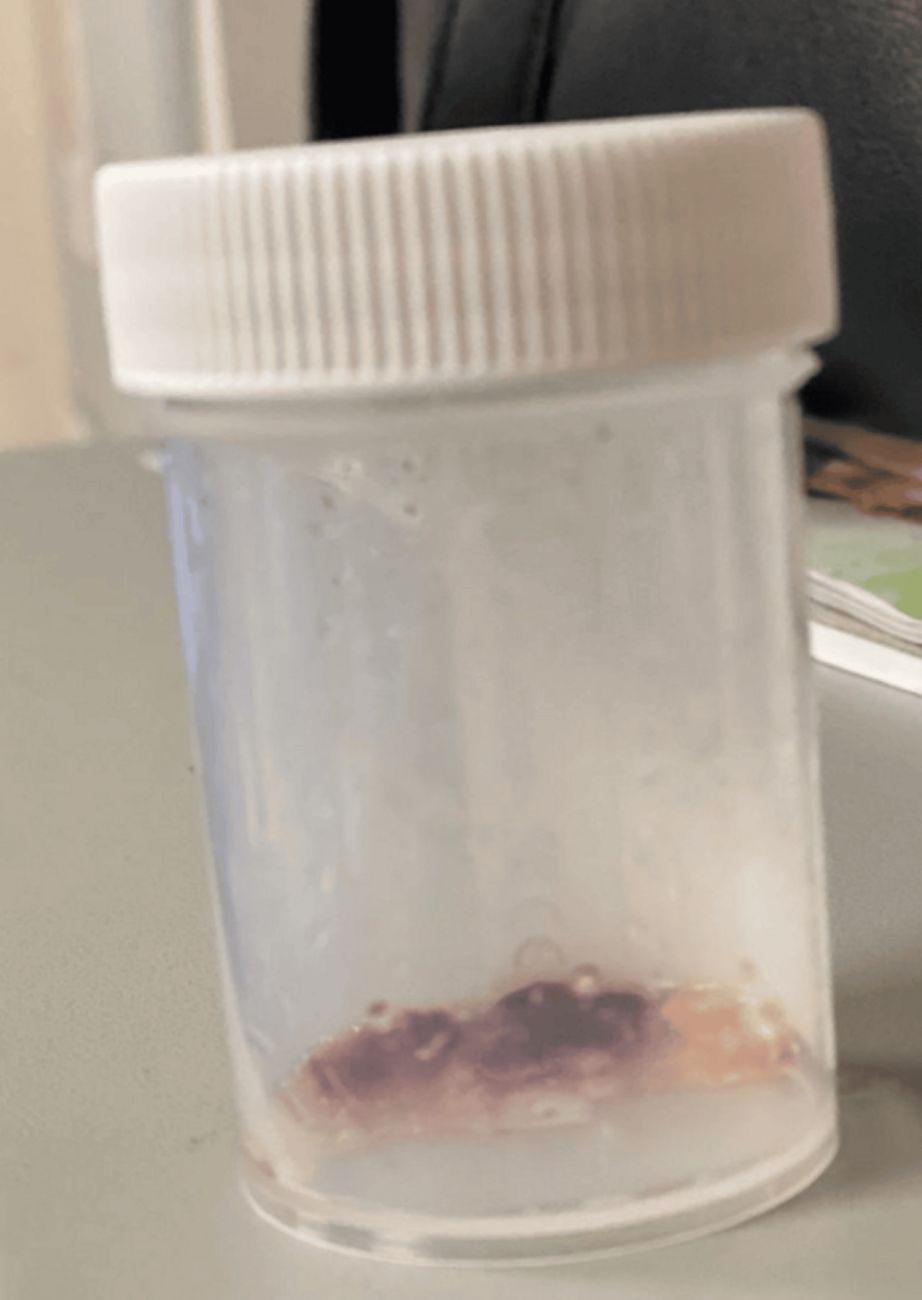
Hemoptysis sample: Recurrent hemoptysis that prompted urgent reassessment, highlighting the severity and progression of pulmonary involvement. Image credit: Hesham K. Badr.

A 90-year-old female presented with a one-month history of productive cough that later progressed to mild, recurrent, progressive hemoptysis, as shown in Figure 1, accompanied by recurrent epistaxis, nasal crusting, fatigue, and general malaise. She was previously active and independent, caring for her husband, until these symptoms began to develop. She reported a noticeable decline in her overall health over the preceding two months, despite previously maintaining a relatively active lifestyle.

In view of her hemoptysis and advancing age, she was referred to the respiratory clinic and was investigated for suspected lung malignancy. She was then sent to the Emergency Department, with vasculitis being a differential diagnosis due to the rapidly progressive presentation.

Along with osteoarthritis, hearing impairment, and a right hip replacement, her past medical history included Stage 4A double-hit B-cell non-Hodgkin’s lymphoma diagnosed in 2017, which was treated with six cycles of R-CHOP chemotherapy (rituximab, cyclophosphamide, doxorubicin, vincristine, and prednisolone), achieving complete remission in 2019.

Upon arrival at the Emergency Department, her observations showed a respiratory rate of 20 breaths per minute, oxygen saturation of 96% on room air, a heart rate of 86 beats per minute, blood pressure of 92/45 mmHg, and a temperature of 36.9 °C. Examination revealed bilateral wheeze with scattered crackles on chest auscultation. Mild pedal edema was noted, with no clinical signs of deep vein thrombosis.

Initial investigations included a full infection workup with chest X-ray (CXR), blood cultures, sputum culture, urine dipstick, viral swabs, and atypical viral screening. CXR revealed bilateral patchy consolidation. Due to elevated CRP count (Table 1) and CXR findings, lower respiratory tract infection was a differential diagnosis, for which she was started on intravenous clarithromycin and co-amoxiclav. It was later escalated to intravenous piperacillin-tazobactam due to persistent symptoms. A renal ultrasound (KUB) excluded obstructive pathology.

Initial blood tests revealed hyperkalemia, elevated urea and creatinine, and a high CRP of 126.4 at the time of presentation in July 2025, which has settled and improved after the treatment (Table 1). A computed tomography (CT) scan of the thorax, abdomen, and pelvis, which was initially done as part of a malignancy workup, revealed multifocal patchy areas of consolidation throughout the lungs, with the largest in the left upper lobe measuring up to 4 cm with surrounding centrilobular ground-glass opacification and air bronchograms, and left thyroid lobe enlargement. 

**Table 1:** Blood markers. eGFR, estimated glomerular filtration rate; CRP, C-reactive protein

Test	September 2025	July 2025	November 2023	September 2020	Reference value
Potassium	4.9	5.6	4.5	4.4	3.5-5.3 mmol/L
Creatinine	151	492	57	67	40-90 µmol/L
Urea	10.1	36.6	4.9	5.3	2.5-7.8 mmol/L
eGFR	26	6	79	72	ml/min/1.73 m²
CRP	3.8	126.4	-	-	0.2-4.9 mg/L

The markedly elevated CRP indicated a significant systemic inflammatory response, while the concurrent rise in serum creatinine and reduction in eGFR demonstrated acute deterioration in renal function. In the context of pulmonary involvement, with increasing oxygen requirements (up to 10 L/minute) and progressive hemoptysis, these findings made a localized infectious process less likely and raised strong concern for an underlying systemic vasculitis. Accordingly, a vasculitis screen was undertaken, as shown in Table 2.

**Table 2:** Autoimmune markers. ds-DNA, double-stranded deoxyribonucleic acid; GBM, anti-glomerular basement membrane; p-ANCA, perinuclear anti-neutrophil cytoplasmic antibody; c-ANCA, cytoplasmic antineutrophil cytoplasmic antibody; RA, rheumatoid arthritis; ELISA, Enzyme-Linked Immunosorbent Assay

Test	July 2025	Reference values
ds-DNA antibody	Negative	Titer U
GBM Quantitation	1.7	0-7 ELISA Units
GBM screen	Negative	Qualitative
Anti-myeloperoxidase (p-ANCA)	1.0	0-3.4 IU/mL ELISA units
ANCA screen (fluorescence)	Positive c-ANCA	IU/mL
Anti-proteinase 3 (c-ANCA)	27.0	0-1.9 ELISA units
Rheumatoid factor-latex screen	31.20	0-20 IU/mL
Complement component C3	1.07	0.70-1.65 g/L
Complement component C4	0.27	0.14-0.54 g/L
RA screen interpretation	Positive	Qualitative
Connective tissue disease screen	Negative	Qualitative

Subsequent testing confirmed PR3-ANCA positivity (Table 2). The markedly elevated PR3-ANCA level strongly supported a diagnosis of GPA, while negative anti-GBM antibodies helped exclude Goodpasture’s syndrome.

The patient was under the care of a geriatric medicine specialist, with involvement from the respiratory and renal teams. She received intravenous methylprednisolone 250 mg, followed by oral prednisolone 30 mg once a day initiated the following day, and was subsequently transferred to the renal ward for further management. She underwent five sessions of plasma exchange and received two doses of intravenous cyclophosphamide (500 mg on July 11 and 500 mg on July 23) and two doses of rituximab (500 mg on July 12 and 500 mg on July 24). Her hemoptysis resolved, and her renal function improved, with serum creatinine decreasing to 151 µmol/L and estimated glomerular filtration rate (eGFR) rising to 26 mL/min/1.73 m² in September 2025 (Table 1).

During her admission, she developed new left leg swelling, and a Doppler ultrasound confirmed an ilio-femoral deep vein thrombosis. Despite receiving venous thromboembolism prophylaxis with enoxaparin as an inpatient, she was switched to apixaban to continue for 6 months as an outpatient with monitoring of renal function tests. She also developed new right-sided hearing discomfort, which was assessed by the otolaryngology team and deemed to be hyperacusis secondary to vasculitic involvement.

She was discharged on a maintenance dose of 5 mg prednisolone once daily, 40 mg omeprazole cover once daily, and avacopan 30 mg twice daily to be continued for one year. She will be followed up as an outpatient by the renal team to review her ongoing steroid maintenance dose. Additional follow-up was arranged with the patient's primary care physician and the relevant specialist teams to monitor renal function, hyperacusis, and treatment response.

## Discussion

This case represents a challenging presentation that required input from multiple teams, multiple investigations, and a prolonged hospital stay due to the uncommon incidence in the patient’s age group and diagnostic considerations. Although not confirmed by biopsy, in light of the patient’s age, the absence of malignancy or other radiological findings, along with positive blood markers, and a satisfactory response to treatment, confirms the diagnosis of GPA, where PR3-ANCA is positive. Other vasculitic conditions, such as EGPA, are associated with similar features, including hemoptysis. However, EGPA is associated with allergies, asthma, and nasal polyps, and the ANCA immunofluorescence pattern is typically perinuclear, with specificity for MPO rather than PR3-ANCA positivity, which is more characteristic of GPA [5]. MPA is another ANCA-associated vasculitis. MPA is more frequently associated with MPO-ANCA and kidney involvement, whereas GPA is more often associated with PR3-ANCA and can have significant lung involvement compared to MPA [6].

GPA is unlikely to occur in old age, and even if present, it would likely manifest between 55 and 75 years of age, but not in the 90s, like our unique case. For example, in 2025, Chevet et al. observed that the majority of cases in their cohort were diagnosed before the age of 60, with only a relatively small *older-onset* group (≥60) included, and no subgroup specifically at the nonagenarian age [7]. An 83-year-old female was identified by Pinckard in 2017 [8]. In 2020, a third case of an 85-year-old man, who presented to the Emergency Department with sudden dyspnea, was recorded by Pace et al. [9]. Other than these cases, others mentioned in the literature are markedly younger in age. In 2020, Lakhani et al. reported a 20-year-old Caucasian male with no past medical history who presented with low-grade fever, left ear pain, and headache for four weeks and was diagnosed with GPA [10]. Another atypical presentation of GPA was reported in 2023 in a 32-year-old male by Maqbool et al. [11]. A challenging case of GPA with cardiac involvement, diagnosed when the patient was only 15 years of age, was reported by Shelton et al. in 2023 [12], and several additional cases across varied age ranges have been reported.

Only a small number of GPA cases have been reported in patients aged 80 years and above. GPA in patients older than 75 remains poorly documented, with sparse data available in those over 80 years old [7]. While Pinckard and Pace et al. described cases in patients aged 83 and 85 years, respectively [8,9]. Reports of GPA in patients aged 90 years or older are exceedingly rare, and to date, no large series describe the diagnostic features, management decisions, or outcomes in nonagenarian patients. This case, therefore, contributes meaningful clinical detail to the very limited literature describing GPA in the ninth decade of life, particularly regarding diagnostic delay and treatment tolerance in frail elderly patients.

Taken together, these reports demonstrate that GPA can present across a broad age range, with considerable variability in clinical features and severity. However, cases occurring in patients aged 90 years and older remain extremely rare, and the clinical presentation may be subtle or initially attributed to comorbidities or age-related decline. Our case, therefore, contributes to the limited literature describing GPA in the very elderly and highlights the importance of maintaining diagnostic vigilance for vasculitis in older adults presenting with pulmonary-renal syndromes.

## Conclusions

The case presented with rapidly progressive hemoptysis, epistaxis, renal impairment, and systemic manifestations that were later complicated by deep vein thrombosis and hearing impairment. Her response to corticosteroids and immunosuppressive medications demonstrates that treatment may achieve benefit even in very elderly patients when carefully monitored. However, vigilance for infection and treatment-related toxicity remains essential. This case highlights the significance of early consideration of ANCA-associated vasculitis in older adults with pulmonary-renal syndromes or unexplained multisystem involvement.

Although GPA most commonly presents between 55 and 69 years of age, onset at ≥90 years is exceptionally rare. In nonagenarians, atypical presentation and diagnostic delay are common due to multimorbidity and initial suspicion of malignancy. This case shows that GPA should still be considered in the differential diagnosis, even in very elderly patients. It also illustrates how prompt ANCA testing and multidisciplinary management can lead to good outcomes and help prevent permanent organ damage.
